# Whose voice counts? Public participation as a legal and commercial determinant of health in South Africa

**DOI:** 10.1136/bmjgh-2025-021501

**Published:** 2026-05-18

**Authors:** Sameera Mahomedy, Nosiphiwo Nzimande

**Affiliations:** 1 SAMRC/Wits Centre for Health Economics and Decision Science (PRICELESS SA), School of Public Health, Faculty of Health Sciences, University of Witwatersrand, Johannesburg, South Africa

**Keywords:** Health policy, Public Health

**SUMMARY BOX**

- Public participation is a constitutional and democratic requirement in South Africa, yet it is rarely analysed as a mechanism through which power, timing and outcomes in health policymaking are structured.
- Drawing on an experience-based vignette and documented policy examples, this commentary demonstrates how administrative delay, limited responsiveness and procedural opacity in participation design shape who can engage and when health-protective policies materialise.
- Reframing public participation as a legal and commercial determinant of health highlights concrete procedural reform levers, including consultation timelines, transparency registers, conflict-of-interest standards and equity-oriented access measures.

## Introduction

South Africa’s constitutional democracy is grounded in principles of openness, accountability and public participation.^1^ These principles are not aspirational—they are binding government obligations, reflected in Section 195 of the Constitution of the Republic of South Africa (1996) and operationalised through various legislation and policy frameworks.^2^ Public participation is particularly salient in South Africa given apartheid and colonialism’s legacy of systematically excluding Black South Africans from decision-making, including decisions that shaped living conditions, health risks and access to care.^3^

In health governance, participation has value beyond democratic legitimacy. Decisions about tobacco and vaping, alcohol control, food control and other non-communicable disease prevention measures determine which risks are prioritised, whose knowledge counts, and how quickly protective interventions are implemented. However, participation does not occur on a level playing field.^4^ Public constituencies are heterogeneous, with different interests and unequal capacity to engage, while organised commercial actors are often better resourced to monitor policy agendas, mobilise submissions and sustain engagement over long time horizons.^5^ Where participation processes are discretionary, opaque or weakly governed, these asymmetries become consequential for policy content and timing. In practice, these asymmetries mean that participation design becomes a site where power is exercised, with implications for whose interests shape health policy outcomes.^6^

This commentary argues that public participation in health policymaking should be understood not only as a democratic and constitutional requirement, but also as a legal and commercial determinant of health.^7 8^ Participation operates as a legal determinant through public law and governance frameworks that structure access, process, and accountability in policymaking, shaping which health risks are prioritised and how protective interventions are designed and implemented. Participation design can also operate as a commercial determinant because poorly regulated participatory spaces can be used strategically to delay, dilute or redirect health-protective policy, especially where transparency and conflict of interest safeguards are limited. Participation is thus not merely a ‘process’ surrounding policy: it is a site of power that shapes what policy becomes, and whether it materialises at all.

We motivate this argument using an experience-based vignette from an attempt to secure interviews with officials across multiple government departments about participation practices in health-related policymaking, alongside documented examples from South Africa and other jurisdictions where the design and governance of consultation processes have intersected with commercial influence. The commentary addresses two questions: (1) What systemic features of participation in policymaking undermine meaningful participation and accountability? (2) What minimum procedural standards could rebalance participation toward transparency, equity and public health protection?

## The gap between legal mandates and practice

While South Africa’s Constitution and various statutes affirm the right to public participation, a persistent gap exists between these legal mandates and their operationalisation in policymaking. A critical distinction arises between legislation-making, which benefits from clear, prescribed procedural requirements and policymaking, where participation processes are often discretionary, weakly regulated, and inconsistently applied.^9^ Unlike legislative processes, where timelines, notice periods and consultation modalities are more clearly defined, policymaking participation frequently lacks equivalent procedural clarity or accountability mechanisms.

This regulatory ambiguity creates uncertainty about what meaningful participation entails in practice and enables participation to become performative rather than substantive. In the absence of clear minimum standards, it is difficult for the public, researchers or civil society to assess whether participatory obligations have been met, how inputs are considered, or how decisions are ultimately justified. These gaps also create conditions in which access to decision-makers may become uneven, informal, or dependent on institutional proximity, resources, or persistence rather than transparent rules. The experience described below provides a concrete illustration of how these governance gaps operate in practice.

## Access challenges: a timeline of delays and challenges

To explore how public participation is operationalised in health-related policymaking, we sought to interview officials across six South African government departments involved in public health policy development (see table 1 for a summary of timelines and issues encountered).

**Table 1:** Timeline of process and communications with the six government departments

Department A (DA)	Department B (DB)	Department C (DC)	Department D (DD)	Department E (DE)	Department F (DF)
19 June 2024 Request email sent to DA	19 June 2024 Request email sent to DB	19 June 2024 Request email sent to DC	19 June 2024 Request email sent to DD	19 June 2024 Request email sent to DE	22 August 2024 Request email sent to DF
8 July 2024 Email received from DA with an internal referral to whom request must be sent	8 July 2024 Follow-up email sent by us	8 July 2024 Follow-up email sent by us	8 July 2024 and 21 August 2024 Follow-up emails sent by us	1 July 2024 Email response received from DE with a referral	26 August 2024 Follow-up sent by us to DF
11 July 2024 Email received from DA with further internal referral to the office of the Director-General (DG)	8 July 2024 Response email received from DB that they have received our email	16 July 2024 Follow-up email sent by us	26 August 2024 Email received from DD with referral	8 July 2024 Follow-up email sent by us	20 September 2024 Email from DF informing us that they have received our request
21 August 2024 Email sent by us with new application to the office of the DG	16 July and 13 August 2024 Follow-up emails sent by us	13 August 2024 Follow-up email sent by us	26 August 2024 Email with new application sent	8 July 2024 Email received from DE with referral	23 September 2024 Email received from DF with documents to initiate process
9 September 2024 Follow-up email sent by us	21 August 2024 New application sent to a different email address	21 August 2024 Follow-up email sent by us	30 August 2024 Follow-up email sent by us	21 August 2024 Email sent to referral email with application	8 October 2024 Email sent to DF submitting documents received
9 September 2024 Email received from DA requiring research application form to be filled out	22 August 2024 Email received from DB stating that they received our application	21 August 2024 Email received from DC informing us that the matter is receiving attention and on the same day, email received from DC with documentation required for security	30 August 2024 Response received from DD that application will undergo vetting process	26 August 2024 and 5 September 2024 Follow-up emails sent to DE	8 October 2024 Email received from DF informing us that request cannot be processed as their system is not functional
25 September 2024 Email sent by us with application form filled out	5 September and 16 September 2024 Follow-up emails sent by us	18 October 2024 Follow-up made by us to the DC	11 follow-up emails sent to request for approval letters (between 16 September 2024 and 31 January 2025)	9 September 2024 Email received from DE informing us that email is received, and internal follow-ups are being done	10 October 2024 Email sent by us enquiring if there are no other processes that can be followed
26 September 2024 Email received from DA informing us that application process takes 2–4 weeks	16 September 2024 Email received from DB with application forms to start process	21 October 2024 Follow-up made by us to DC	31 January 2025 Email received from DD stating that once approval is granted, they will contact us to let us know	9 follow-up emails sent but no response (between 2 October and 24 January 25)	3 follow-up emails sent but no response (between 13 November 2024 and 14 January 2025)
8 October 2024 Email with approval letter received from DA	25 September 2024 Email sent by us with application forms	21 October 2024 Email received from DC informing us that matter is undergoing internal vetting			17 January 2025 Email received from DF with consent form and enquiring on availability for fingerprint taking
3 follow-up emails for interviews sent (between 13 November 2024 and 20 January 2025)	02 October 2024 Email received from DB asking for clarity on what the study is for	2 follow-up emails sent for study approval (4 November and 13 November 2024)			20 January 2025 Email received from DF enquiring availability for fingerprint taking
30 January 2025 Interview with DA official held	18 October 2024 New application sent by us to DB	13 November 2024 Approval letter granted			20 January 2025 Email sent by us enquiring what the fingerprints are for and explaining that an email had been received on 17 January 2025 with the consent form which was being completed
18 follow-up emails for second interview sent but no response (between 25 February and 6 May 2025)	7 follow-up emails sent before approval of study granted (between 4 November 2024 and 23 Jan 2025)	10 follow-up emails sent to secure interviews but no response (between 13 November 2024 and 19 March 2025)			21 January 2025 Email received from DF requesting contact details so that they can provide further clarity
	23 January 2025 Approval letter for study granted				21 January 2025 Phone call received from DF explaining the fingerprint process, enquiring on the study and further explaining that they only undertake student research
	18 follow-up emails sent requesting details for officials to be interviewed but no response (between 23 January and 23 April 2025)				21 January 2025 Email received from DF confirming telephonic conversation and informing us that only student research is undertaken by them
Status to date: Permission letter received and one interview conducted	Status to date: Permission letter received, no interviews conducted	Status to date: Permission letter received, no interviews conducted	Status to date: Awaiting permission letter	Status to date: No permission letter	Status to date: No permission letter

Despite initiating the process in June 2024 and complying with evolving procedural requirements, permission to conduct interviews was repeatedly delayed over many months. The first permission letter was received approximately 4 months after initial contact (after a multitude of follow-ups), with subsequent approvals following several months later. At the time of writing, permission has still not been granted by three departments despite repeated follow-up attempts, and only one interview has been completed due to ongoing non-responsiveness. This experience illustrates how extended timelines, limited feedback and procedural uncertainty can constrain access to policymakers responsible for shaping public health policy, even when formal participation channels are followed.

## Why this matters: beyond legal formalities

Barriers to accessing policymakers are not merely procedural inconveniences, they also have material implications for public health governance, democratic legitimacy and equity. When participation is limited, delayed, or rendered opaque, those most affected by health policies, particularly communities already burdened by structural inequalities, are least able to shape policy agendas or scrutinise decision-making processes. At the same time, participation asymmetries are amplified where engagement rules are discretionary, poorly specified or inconsistently applied, advantaging actors with greater resources and sustained access to policymaking processes.

These dynamics are evident in several South African health policy processes and internationally where the design and governance of participation mechanisms have intersected with sustained industry engagement and policy delay (see table 2). In the case of the Health Promotion Levy (sugar-sweetened beverage tax), beverage industry actors actively participated in consultation processes, advancing economic impact narratives that contributed to the adoption of a lower than recommended tax rate and subsequent policy stagnation. Similarly, the front-of-pack warning labels process illustrates how, despite the public comment period closing in September 2023, the absence of clear procedural timelines and public disclosure of how submissions are considered or when decisions will be taken can limit accountability and contribute to prolonged policy uncertainty in an area of urgent public health need.^10^ Although these examples do not imply that participation processes are solely responsible for policy delay, they illustrate how weak procedural safeguards can shape the pace and substance of health regulation and act as a legal determinant.

**Table 2:** Selected South African and global examples illustrating how consultation processes have been used to delay or dilute health policy interventions

Country	Policy/issue	Nature of delay or obstruction	Key actors involved	Notes	Participation mechanism implicated	References
South Africa	Health Promotion Levy (2016–present)	Industry lobbying, concerns about economic impact, need for further consultation	Beverage companies, business lobby groups	Despite the initial policy proposal recommending a 20% tax, industry relied on job loss arguments to oppose the tax. Consequently, the tax was implemented at a lower rate and has not been increased for inflation since implementation.	Economic framing in participation process; prolonged consultation/administrative delay	Abdool Karim S, Kruger P, Hofman K. Industry strategies in the parliamentary process of adopting a sugar-sweetened beverage tax in South Africa: a systematic mapping. Global Health 2020; 16(116). doi: 10.1186/s12992-020-00647-3.
South Africa	Front-of-pack labelling (2022–present)	Multiple iterations. Delay in reviewing comments submitted	Unclear due to public comments not being made available	Draft regulations published for public comments in May 2023. Deadline was extended with final commenting period closed in September 2023. To date, comments have not been made public, nor is there any clarity on when the regulations will be finalised.	Non-disclosure of submissions or decision rationales; prolonged consultation/administrative delay	Radu YT, Mahomedy S. Front-of-package labelling: A public health imperative rooted in the right to health. Int J Equity Health 2025; 24(116). doi: 10.1186/s12939-025-02473-8.^10^
South Africa	Tobacco Products and Electronic Delivery Systems Control Bill (2022–present)	Delay in introducing the Bill to parliament and further delays in implementing the Bill due to tobacco industry interference	Tobacco industry	Approximately a 4.5-year delay in implementing the Bill due to interference by tobacco industry including sponsorship of organisations that work closely with government.	Litigation threat/legal uncertainty; prolonged consultation/administrative delay	Moodley V. South Africa Tobacco Industry Interference Index [online]. 2023.(accessed on 15 July 2025).
South Africa	Liquor Amendment Bill (2016–present)	Alcohol industry lobbying and interference	Alcohol industry	Alcohol industry lobbying and interfering with the process through political party lobbying, providing alternative policy and further delaying implementation of the regulations.	Economic framing in participation process; prolonged consultation/administrative delay	Mitchell G, Siwela P, Goldstein S, Diedericks AM. Alcohol industry involvement in the delayed South Africa Draft Liquor Amendment Bill 2016: A case study based on freedom of information requests. Global Health 2025; 21(1):11. doi: 10.1186/s12992-025-01097-5.
Australia	Plain Packaging of Tobacco (2008–2012)	Legal threats and lobbying by tobacco industry delayed final implementation	British American Tobacco, Philip Morris	Industry used trade agreements and lawsuits to challenge the law; delayed uptake in other countries.	Litigation threat/legal uncertainty; prolonged consultation/administrative delay	Cohen JE, Zhou S, Goodchild M, Allwright S. Plain packaging of tobacco products: Lessons for the next round of implementing countries. Tob Induc Dis 2020; 18(94). doi: 10.18332/tid/130378.^11^
Mexico	Sugary Drink Tax (2013)	Industry lobbying delayed legislative support and tried to weaken tax rate	Mexican Beverage Association (ANPRAC), Coca-Cola	Despite WHO support, industry framed tax as harmful to the poor and small businesses.	Economic framing in participation process; prolonged consultation/administrative delay	Pedroza-Tobias A, Crosbie E, Mialon M, Carriedo A, Schmidt LA. Food and beverage industry interference in science and policy: efforts to block soda tax implementation in Mexico and prevent international diffusion. BMJ Glob Health 2021;6 (8). doi: 10.1136/bmjgh-2021-005662.
Kenya	Tobacco Control Bill (1999–2007)	Bill remained with Attorney General for 8+ years due to industry pressure	British American Tobacco Kenya	Close industry ties to politicians enabled direct influence over delays and dilution.	Prolonged consultation/administrative delay; litigation threat/legal uncertainty	Mohamed SF, Juma P, Asiki G, Kyobutungi. Facilitators and barriers in the formulation and implementation of tobacco control policies in Kenya: a qualitative study. BMC Public Health 2018; 18(960). doi: 10.1186/s12889-018-5830-x.
Philippines	Tax on Sweetened Beverages (2017–2018)	Delay and weakening of the tax during legislative process due to beverage industry lobbying	Beverage companies, business lobby groups	Originally broader health tax was narrowed to exclude certain products.	Economic framing in participation process	Huse O, Backholer K, Nguyen P *et al* A comparative analysis of the cost-utility of the Philippine tax on sweetened beverages as proposed and as implemented. Lancet Reg Health West Pac 2023; 41 (100912). doi: 10.1016/j.lanwpc.2023.100912
Brazil	Ultra-Processed Food Labelling (2014–2020)	Labelling policy delayed for 6 years after strong resistance from powerful food industry groups	Brazilian Food Industry Association	Civil society eventually succeeded in getting warning labels approved in 2020.	Prolonged consultation/administrative delay; economic framing in participation process	Mais LA, Borges CA, Khandpur N, Duran AC, Martins APB. Brazil’s nutrition labelling regulation: Challenges ahead on the path to guaranteeing consumer’s right to adequate information. Front Nutr 2022; 9 (921519). doi: 10.3389/fnut.2022.921519.
USA	E-cigarette Regulation (2016–2023)	Regulatory loopholes and extended deadlines allowed industry to delay oversight for nearly a decade	JUUL, tobacco industry, FDA	Youth vaping surged in the interim due to lack of timely regulation.	Litigation threat/legal uncertainty; prolonged consultation/administrative delay	Watts C, Rose S, McGill B, Yazidjoglou. New image, same tactics: global tobacco and vaping industry strategies to promote youth vaping. Health Promot Int 2024; 39(5). doi: 10.1093/heapro/daae126.
UK	Advertising Restrictions on Junk Food (2019–January 2026)	Multiple postponements of planned advertising bans on unhealthy food products	Food and advertising industries	Government cited economic challenges, but critics flagged lobbying and industry pressure.	Prolonged consultation/administrative delay	Gregory A. UK ban on junk food adverts targeting children is delayed until next year [online]. 2025. (accessed 15 July 2025). Donnelly L. Ministers ‘caving in to food industry’ after delaying junk food ads ban [online]. 2025. (accessed 16 July 2025)

ANPRAC, Asociación Nacional de Productores de Refrescos y Aguas Carbonatadas (now MexBeb - Asociación Mexicana de Bebidas); FDA, Food and Drug Administration.

Comparable patterns have been documented globally. Australia’s plain packaging reforms, for example, demonstrate how extended consultation, legal threats, and procedural contestation were used to delay implementation, even in the presence of strong public health evidence.^11^ Across these contexts, participation processes themselves become sites of policy contestation rather than neutral mechanisms for inclusive deliberation. Through these cases, recurring forms of interference through participation channels can be observed, including prolonged consultation and administrative attrition; limited disclosure of submissions or decision rationales; economic framing that recasts health regulation as a threat to growth or employment; and the use of legal uncertainty to deter timely policy action.

## Addressing the participation paradox: from tick-box to a determinant of health

Public participation already functions as a legal and commercial determinant of health by shaping who can influence policy agendas, how decisions are made, and whose interests ultimately prevail. The question, therefore, is not whether participation matters, but how it is governed. Participation processes that are discretionary, opaque, or weakly regulated risk entrenching power asymmetries and enabling policy delay or dilution rather than promoting accountability and equity.

At minimum, participation in health policymaking should be underpinned by clearly articulated procedural safeguards aligned with existing governance instruments, including South Africa’s National Policy Development Framework. These standards should include:

1. Clearly defined notice periods and timelines for consultation, decision-making, policy finalisation;
2. Mandatory public disclosure of submissions, meetings, and decision rationales following consultation; and
3. Clear points of accountability where delays or deviations from process occur.

Such measures would directly address the administrative attrition and opacity observed across multiple policy processes.

To mitigate undue commercial influence, participation frameworks should also incorporate robust conflict of interest safeguards, drawing on established public health governance norms such as the WHO Framework Convention on Tobacco Control Article 5.3.^12^ This includes disclosure of stakeholder engagements, limits on industry participation in policy design, and transparency around how commercial submissions are weighed relative to public health evidence. Several jurisdictions including the United Kingdom and the Philippines have operationalised Article 5.3 through domestic rules that restrict industry engagement and require disclosure of interactions in health policymaking, illustrating feasibility of such safeguards in practice.

Finally, participation standards must be explicitly equity oriented. Digital-only submissions, technical language, short notice periods, and weekday hearings disproportionately exclude rural communities, low-income groups, persons with disabilities, youth, and community-based organisations.^13^ Addressing these barriers requires practical measures, such as language access, non-digital submission options, and community-level engagement mechanisms, so that participation does not reproduce the very inequities health policies seek to address. Together, these reforms reposition participation from a procedural formality to a substantive governance function capable of shaping timely, transparent, equitable health policy.

## Conclusion

Public participation already operates as a legal and commercial determinant of health by shaping who can access decision-makers, how policy debates unfold and whether health-protective measures are advanced, delayed or diluted. The experience described in this commentary illustrates how administrative opacity, prolonged timelines and limited feedback can constrain meaningful participation in health policymaking, even where formal participation channels exist. When such conditions persist, participation risks becoming performative rather than substantive, undermining both democratic accountability and public health outcomes.

Strengthening participation in health policymaking does not require the creation of entirely new institutions. Rather, it requires clearer procedural standards, greater transparency and deliberate safeguards against structural power asymmetries. Within the next 12–18 months, governments could take three concrete, monitorable steps. First, mandate minimum procedural timelines for consultation, decision-making and policy finalisation, alongside requirements to publish public submissions and reasoned responses. Second, introduce conflict of interest disclosure and engagement registers for health policymaking, drawing on established public health governance norms to ensure transparency around commercial participation. Third, use inclusive participation mechanisms such as language access, non-digital submission options and community-level engagement to ensure that participatory processes do not systematically exclude already marginalised groups.

In contexts where health policies are contested and delays carry real population health costs, treating participation as a determinant of health clarifies what is at stake when access, transparency and accountability are allowed to erode.

## Data Availability

No datasets are publicly available. This commentary is based primarily on reflections arising from an attempted qualitative study and related engagement processes. No identifiable participant data are reported.
